# Spatial and Temporal Dynamics of Lymphocytic Choriomeningitis Virus in Wild Rodents, Northern Italy

**DOI:** 10.3201/eid1507.081524

**Published:** 2009-07

**Authors:** Valentina Tagliapietra, Roberto Rosà, Heidi C. Hauffe, Juha Laakkonen, Liina Voutilainen, Olli Vapalahti, Antti Vaheri, Heikki Henttonen, Annapaola Rizzoli

**Affiliations:** Edmund Mach Foundation–Istituto Agrario di San Michele all’Adige, San Michele all’Adige, Italy (V. Tagliapietra, R. Rosà, H.C. Hauffe, A. Rizzoli); Finnish Forest Research Institute, Vantaa, Finland (J. Laakkonen, L. Voutilainen, H. Henttonen); University of Helsinki, Helsinki, Finland (J. Laakkonen, O. Vapalahti, A. Vaheri)

**Keywords:** Lymphocytic choriomeningitis virus, arenavirus, viruses, Italy, wild rodents, zoonoses, research

## Abstract

We determined the prevalence of infection with lymphocytic choriomeningitis virus (LCMV) among small mammals in northern Italy and analyzed long-term dynamics of LCMV in a rodent population in the province of Trento. LCMV is circulating among the most widespread and common wild rodent species in this area (*Apodemus flavicollis*, *Myodes glareolus*, and *Microtus arvalis*); overall prevalence is 6.8%. During 2000–2006, intensive monitoring of LCMV in a population of yellow-necked mice (*A. flavicollis*) showed a positive correlation between prevalence of infection and rodent density. At the individual level, weight and sex appeared to correlate with antibody prevalence, which suggests that horizontal transmission of LCMV occurs principally among heavier, older males and occurs during fighting. Isolation and genetic characterization of this virus will be the crucial next steps for a better understanding of its ecology.

## Abstract 2

Prevalence of virus infection was higher in areas of greater rodent density.

Viral hemorrhagic fevers caused by arenaviruses pose serious human public health risks and cause devastating and often lethal disease. These diseases include Lassa hemorrhagic fever in West Africa, Junín hemorrhagic fever in Argentina, Machupo and Chapare hemorrhagic fevers in Bolivia, Guanarito hemorrhagic fever in Venezuela, and Sabià hemorrhagic fever in Brazil. In recent years, increased air travel between Africa and other continents led to the importation of cases of Lassa fever virus into the United States, Europe, Japan, and Canada and caused increasing concern about the potential of arenaviruses to trigger new emerging disease foci (*1*–*3*).

Lymphocytic choriomeningitis virus (LCMV) is a rodent-borne arenavirus (family *Arenaviridae*, genus *Arenavirus*) first reported in St. Louis, Missouri, USA, in 1934 (*4*). It is primarily associated with the house mouse (*Mus musculus*) (*5*); prevalence rates among this species range from 2.5% to 9.0% in the United States (*6*,*7*), 11.7% in Spain (*8*), 3.6% in Germany (*9*), and 7.0% in Japan (*10*).

LCMV commonly infects T cells in house mice, and these animals may act as carriers with long-term or life-long viremia and viruria and negligible signs of acute disease (*5*,*11*). Several wild rodent species are seropositive for LCMV: *Mus spretus* (Algerian mouse), *Apodemus agrarius* (striped field mouse), *A*. *flavicollis* (yellow-necked mouse), *A*. *sylvaticus* (wood mouse), *A*. *mystacinus* (eastern broad-toothed field mouse), *Micromys minutus* (harvest mouse), *Microtus levis* (syn. *M*. *rossiaemeridionalis* [sibling vole]), *Chionomys roberti* (Robert’s snow vole), *Myodes glareolus* (bank vole), and *Arvicola scherman* (montane water vole) (*8*,*12*–*15*). Hamsters and guinea pigs may also become infected after close contact with infected *M*. *musculus* mice and may also be asymptomatic (*16*). LCMV transmission in natural rodent hosts occurs vertically, horizontally, and during sexual intercourse. However, horizontal and vertical transmission may lead to different outcomes. Horizontal transmission may cause only transient viremia, and vertical transmission may cause chronic infection (*17*,*18*).

Humans become infected with LCMV by inadvertently inhaling aerosolized rodent excreta or secreta (*7*,*8*). Human-to-human transmission has not been reported, except for 1 case of vertical transmission from an infected mother to her fetus (*19*). LCMV-infected humans are generally asymptomatic or show mild influenza-like symptoms. However, LCMV infection can also lead to aseptic meningitis, meningoencephalitis, and congenital abnormalities (*20*). Immunocompromised persons are particularly susceptible to infection with LCMV; deaths caused by LCMV in organ transplant recipients have been reported (*21*).

Although LCMV is the only arenavirus reported in Europe (*12*), data on its incidence and epidemiologic features on this continent are insufficient. Only antibodies against LCMV in humans have been reported from Spain (1.7%) (*8*) and the Netherlands (2.9%) (*22*). In 2002, a preliminary survey of LCMV in the province of Trento in northern Italy showed an antibody prevalence of 5.6% for wild rodents (6.1% for *A*. *flavicollis*, 3.3% for *M*. *glareolus*, and 14.3% for *Microtus arvalis* [common vole]) and 2.5% for forestry workers (*14*). The occurrence of LCMV or LCMV-related viruses in several rodent species in Europe has led to the suggestion that LCMV could represent a complex of strains or closely related arenaviruses hosted by different rodent species (*12*,*14*).

We studied the distribution and prevalence of LCMV in small mammals throughout the Alps in northern Italy. Moreover, we analyzed the dynamics of LCMV in an intensive, long-term, capture-mark-recapture study of rodents in the province of Trentino in Italy. We also studied whether patterns of pathogen prevalence vary at the population level (density, season, time, space) or individual level (weight, sex, and breeding status).

## Materials and Methods

### Study Sites

**Figure 1:**
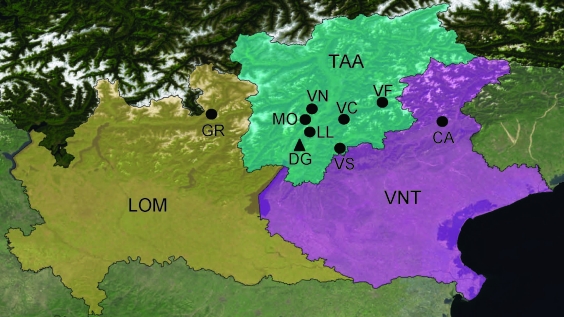
Study sites for trapping of rodents and isolation of lymphocytic choriomeningitis virus in Lombardy (LOM), Trentino-Alto Adige (TAA), and Veneto (VNT) in northern Italy, 2000–2006. GR, Grosotto/Mazzo; MO, Molveno; VN, Val Non; VC, Val Cembra; VF, Val Fiemme; LL, Laghi Lamar; DG, Dos Gaggio; VS, Val Sella; CA, Candaten. Circles indicate sites of extensive sampling and triangles indicate sites of intensive sampling. Background map: True marble by Unearthed Outdoors LLC (Madison, WI, USA) is licensed under a Creative Attribution 3.0 United States License (www.unearthedoutdoors.net/global_data/true_marble/download).

Extensive sampling was conducted during 2002–2006 at 8 sites in northern Italy (Figure 1): 1 in Lombardy (province of Sondrio), 1 in Veneto (province of Belluno), and 6 in Trentino-Alto Adige (province of Trento). Intensive monitoring was conducted during 2000–2006 in Valle dei Laghi, province of Trento, in the northeastern Italian Alps (Dos Gaggio, Municipality of Cavedine, 50°56′15′′N, 16°31′13.8′′E) (Figure 1). This site is located on an isolated calcareous ridge (750–800 m above sea level), is dominated by broadleaf forest (*Fagus sylvaticus*, *Carpinus betulus*, *Fraxinus ornus*, *Corylus avellanae*), and includes plantations of larch (*Larix decidua*), spruce (*Picea abies*), and pine (*Pinus sylvestris, P*. *strobus*). Forest management includes coppices and coppices converted to high-stand forest. Small meadows (<1 hectare) are scattered throughout the woodland.

### Rodent Monitoring

Extensive samples were obtained at the 8 sites. Of these sites, 6 were surveyed in 2002 during 1 session of 4 days and 3 nights, and 3 were surveyed in 2006 during 4 sessions of 4 days and 3 nights. Multicapture live traps (Ugglan Special Mouse Trap 2; Grahnab, Hillerstorp, Sweden) were used to capture animals. Live-trapped rodents were subsequently killed with isofluorane. During necropsy, samples from lungs, spleens, and kidneys were collected and stored at –80°C until analysis.

During 2000–2006 at Dos Gaggio, rodents were intensively live-trapped by using capture-mark-recapture techniques and Ugglan live traps (Grahnab). Trapping was conducted every 2 weeks for 2 consecutive nights from April through October and occasionally from November through March on 8 × 8 grids (64 traps) with a 15-m distance between traps. Nine grids were used during 2000–2002, and 4 grids were used during 2003–2006. At first capture, a passive-induced transponder (ID 100; Trovan, Hessle, UK) was implanted subcutaneously into each animal. Species, sex, breeding condition, and weight were recorded.

Because small rodent populations are seasonally and multiannually heterogeneous, individual rodents were categorized into the following functional groups (*23*–*25*): juveniles, <1 month of age, gray pelage [fur], and weight <15 g); subadults, weight >15 g and not in breeding condition (undescended testes or imperforate vagina); and adults, weight >15 g and in breeding condition (descended testes or perforate vagina, visible nipples, or visibly pregnant). At the end of the breeding season, some postbreeding adults may by definition appear to be subadults. To ensure that postbreeding animals were not included in the subadult category, animals that were defined as adults once during the season were considered adults for all statistical analyses. Blood samples were collected once per 2-week trapping session from the suborbital venous plexus by using a microhematocrit capillary tube (length 75 mm, diameter 1.15 mm). Blood samples were centrifuged and serum samples were stored at –20°C until analysis.

### Rodent Densities

Population density of yellow-necked mice (*A*. *flavicollis*) at intensively studied sites was estimated by using the Jolly-Seber mark–recapture model (*26*). Population density at extensive sites was determined by calculating the small mammal abundance index (SAI) according to the equation SAI = (SC × 100) / (T × N), in which SC is the number of rodents captured, T is the number of traps, and N is the number of nights).

### Antibody Assays

All serum samples were tested for immunoglobulin G against LCMV by using an indirect immunofluorescent antibody assay as described (*12**,**27*). The LCMV strain used in this assay was obtained from the Swedish Institute for Infectious Disease Control (Stockholm, Sweden). Animals that were positive or weakly positive for LCMV and animals that were negative after showing a positive result at a previous trapping session were retested when possible. LCMV-positive animals were assumed to have a chronic infection.

### Statistical Analyses

To assess spatial differences in seroprevalence of LCMV at many sites, we used a generalized linear model with a binomial error and S-PLUS version 7.0 software (TIBCO Software Inc., Palo Alto, CA, USA). The binary response variable was the presence or absence of virus antibodies in mouse serum samples, and explanatory variables were province; trapping site; rodent weight, sex, and breeding status by species; rodent abundance index; and trapping year.

For the intensive dataset for Dos Gaggio, only data for *A*. *flavicollis* mice were analyzed (at population and individual levels) because other rodents of other species were rarely trapped at this site and none were infected with LCMV. To assess whether antibodies against LCMV in *A*. *flavicollis* mice were affected by any host or population characteristics, we used generalized linear mixed models (GLMMs) with a penalized quasilikelihood algorithm and binomial and S-PLUS version 7.0 software. In this analysis, the presence of antibodies against LCMV was the response variable. For population analysis, rodent density and trapping month and year were the explanatory variables. For individual analysis, sex, breeding status, and weight were selected to identify the model that best explained variance in the presence of virus antibodies.

To overcome autocorrelations caused by multiple trapping of the same rodent, the unique transponder code of each animal was entered into GLMMs as a random effect. Variance explained by each explanatory factor and levels of significance were calculated by using a stepwise backward deletion test (*28*).

## Results

During 99,464 trap nights (9,864 in extensive monitoring and 89,600 in long-term intensive monitoring), 2,342 rodents in 5 species (*A*. *agrarius*, *A*. *flavicollis*, *A*. *sylvaticus*, *M*. *glareolus*, and *M*. *arvalis* were trapped. *A*. *flavicollis* and *M*. *glareolus* were the most frequently trapped species (87.6% and 5.7%, respectively). A total of 3,215 serum samples (2,732 at Dos Gaggio and 483 in the extensive sampling) were analyzed.

### Extensive Sampling

The overall prevalence of LCMV was 8.3% (40/483) (Table). Antibodies were detected in all species except *A*. *agrarius* and *A*. *sylvaticus*. The highest prevalence was in *M*. *arvalis* voles (20%), although sample size for this species was low (n = 5). For the more abundant and ubiquitous species (*A*. *flavicollis* and *M*. *glareolus*); prevalence was 8.9% and 7.4%, respectively.

**Table:** Prevalence of lymphocytic choriomeningitis virus in 5 rodent species at extensive trapping sites, northern Italy, 2002 and 2006*

Region	Province	Site	Rodent species					Total no. rodents	Seroprevalence, % (no. positive/ no. tested)
			*Apodemus agrarius*	*A*. *flavicollis*	*A*. *sylvaticus*	*Myodes glareolus*	*Microtus arvalis*		
VNT	BL	Candaten	2	44	1	5	0	52	3.8 (2/52)
LOM	SO	Grosotto/Mazzo	0	49	0	32	0	81	9.9 (8/81)
TAA	TN	Val Cembra	0	97	4	26	5	132	8.3 (11/132)
TAA	TN	Val Fiemme	0	11	0	7	0	18	5.5 (1/18)
TAA	TN	Laghi Lamar	0	63	0	34	0	97	3.1 (3/97)
TAA	TN	Molveno	0	30	0	1	0	31	12.9 (4/31)
TAA	TN	Val Non	0	35	0	30	0	65	13.8 (9/65)
TAA	TN	Val Sella	0	7	0	0	0	7	28.6 (2/7)
Total†			2 (0)	336 (8.9)	5 (0)	135 (7.4)	5 (20)	483 (8.3)	

*VNT, Veneto; BL, Belluno; LOM, Lombardy; SO, Sondrio; TAA, Trentino-Alto Adige; TN, Trento.

The seroprevalence rate was highest in Val Sella (28.6%; 2/7), but the number of samples was low. The province of Belluno in the region of Veneto had a prevalence of 3.8%. Seroprevalence rates in Sondrio (region of Lombardy) and Trento (region of Trentino-Alto Adige) were 9.9% and 8.8%, respectively. None of the explanatory variables of the extensive dataset (province, trapping site, species, sex, weight, breeding status, rodent abundance index, trapping year) affected the presence of antibodies against LCMV in the rodent species sampled, except in *A*. *flavicollis* mice.

### Temporal Dynamics of LCMV in *A. flavicollis* Mice

**Figure 2:**
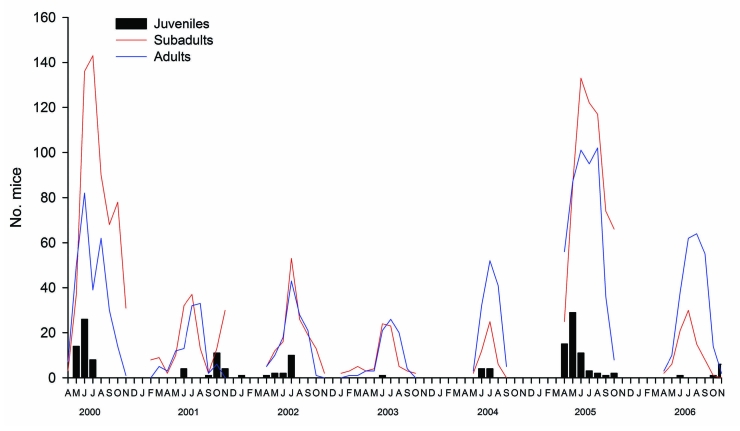
Population structure of *Apodemus flavicollis* in Dos Gaggio region of Trentino Alto-Adige, northern Italy, 2000–2006. Juveniles, <1 month of age, gray pelage (fur), and weight <15 g); subadults, weight >15 g and not in breeding condition (undescended testes or imperforate vagina); adults, weight >15 g and in breeding condition (descended testes or perforate vagina, visible nipples, or visibly pregnant). Gaps in the plots indicate that no trapping was conducted during these periods.

During 2000–2006 (89,600 trap nights) at Dos Gaggio, 1,717 yellow-necked mice were trapped and 7,523 recaptures were reported. This population shows a multiannual and seasonal variation; the population density begins to increase in the spring (end of February through March), marking the beginning of the reproductive period; reaches a peak in mid-summer (end of July through the end of August); and is followed by a decrease during the winter months. In 2001 and 2005, a second peak occurred in autumn. The years with the highest density were 2000 (10.78 animals/hectare) and 2005 (17.03 animals/hectare), both of which followed a year of extensive seed production (masting) of beech trees at this site, which apparently favored rodent survival and prolonged the mating season into the winter months. The population structure (Figure 2) showed a large number of juveniles captured in the spring of high-density years that followed years of extensive seed production, which confirmed that mouse reproduction extended into the intervening winter.

A total of 205 (7.5%) of 2,732 serum samples from *A*. *flavicollis* mice contained antibodies against LCMV. The prevalence per year ranged from 0.97% to 14.1%; rodent populations had the highest seroprevalence in 2002 (14.14%, 57/403), followed by 2005 (9.88%, 113/1144), 2006 (5.36%, 17/317), 2003 (5.04%, 7/139), 2004 (2.73%, 5/183), 2000 (1.18%, 4/389), and 2001 (0.97%, 2/207).

**Figure 3:**
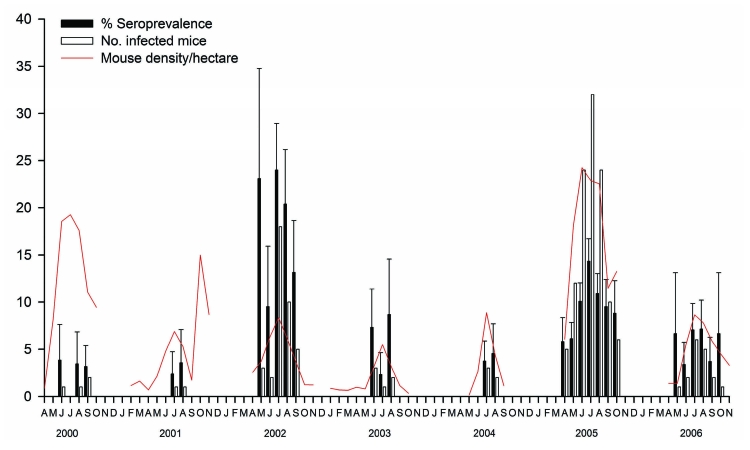
Correlation between dynamics of arenavirus seroprevalence, number of infected rodents, and density of *Apodemus flavicollis* in Dos Gaggio region of Trentino Alto-Adige, northern Italy, 2000–2006. Error bars indicate standard errors. Gaps in the plots indicate that no trapping was conducted during these periods.

**Figure 4:**
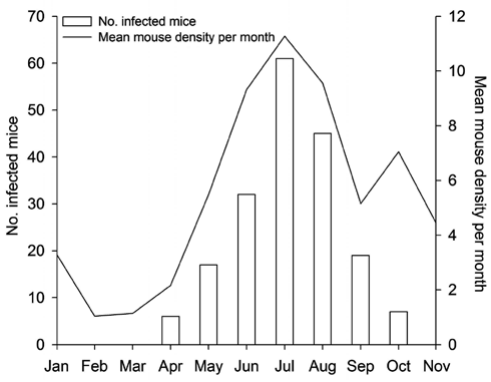
Monthly number of lymphocytic choriomeningitis virus–positive animals and mean rodent density per month (pooled data), northern Italy, 2000–2006.

The model that best explains the difference in LCMV seroprevalence at the population level includes trapping year and rodent density. Specifically, model coefficients showed a significant difference in prevalence among trapping years (F_6,1579_ = 15.13, p<0.001) and a positive correlation with rodent density (F_1,1579_ = 68.36, p<0.001) (Figures 3, 4).

At the individual level, seroprevalence showed a positive correlation with weight (F_1,1545_ = 240.04, p<0.001). Juvenile and subadult mice were significantly less likely to be infected than adults (F_2,1545_ = 101.1, p<0.001). LCMV seroprevalence was not influenced by sex (F_1,1545_ = 0.04, p = 0.82), but sex and weight produced a significant interaction; stronger positive effect of weight on LCMV seroprevalence was stronger for male rodents than for female rodents (F_1,1545_ = 4.57, p = 0.032).

## Discussion

The prevalence and transmission rates of rodent-borne viruses in host populations vary in time and space and among host–virus systems. Improving our understanding of the causes of these variations will lead to a better understanding of changes in disease risk to humans. Our study highlights the endemicity of LCMV (or LCMV-related viruses) in small rodents in northern Italy and complements reports of LCMV seroprevalence in humans in Italy (*14*).

Our results show that LCMV or LCMV-related viruses are circulating among the most widespread and common wild rodent species other than the typical reservoir (*M*. *musculus*). These species are *Microtus arvalis* (prevalence 20%), *A*. *flavicollis* (8.9%) and *M*. *glareolus* (7.4%) (Table). Only *A*. *agrarius* and *A*. *sylvaticus* mice were not positive for LCMV antibodies. These results confirm those of previous studies (*14*,*15*), which detected antibodies against LCMV in rodent species of the subfamily Arvicolinae. Additional arenaviruses may be present in Europe.

Overall prevalence of LCMV among rodents in our study, including results from extensive and intensive sampling sites, was 6.8%, which is comparable to that reported in the study of Kallio-Kokko et al. (*14*) (5.6%). All of our sites had rodents positive for LCMV; prevalences were higher in the provinces of the central Alps (8.8% in Trento and 9.9% in Sondrio) than in Belluno in the eastern Alps (3.8%). Because all trapping grids were set in similar habitats, the density and diversity of rodent species were comparable between provinces. LCMV appears to be less common in the eastern Alps than in the central Alps. Further investigation is needed to determine the reasons for this difference.

The long-term intensive trapping system used at Dos Gaggio provided a unique opportunity to document the dynamics of LCMV in a rodent community dominated by *A*. *flavicollis* mice. Use of GLMMs provided a powerful tool for overcoming nonindependence of data resulting from repeat samples taken from the same rodent. Our analysis showed that mean annual population density showed a correlation with prevalence of infection in *A*. *flavicollis* mice (Figure 3). This result is consistent with what is known about the behavior of this species. Increases in density also increase overlap between neighboring home ranges, the number of contacts, and conflict between rodents and thus increase the potential for virus transmission (*29*,*30*). Two exceptions are evident in the 2 years of high density of rodents (2000 and 2005). In 2000, LCMV prevalence was particularly low, but low prevalence may have been caused by the low number of samples analyzed (20% of the total). The low prevalence finding in 2005 could have been caused by the large proportion of juveniles captured, which, as our data indicate, tend to be LCMV negative and would lower overall prevalence.

Sporadic production of mast-producing trees, such as beech, is an important environmental factor that affects the dynamics of many forest rodents in temperate Europe. Mast-driven outbreaks in bank voles (*M*. *glareolus*) in Belgium have led to outbreaks of nephropathia epidemica, a mild form of hemorrhagic fever with renal syndrome caused by Puumala hantavirus (*31*). Our results imply that masting also affects the dynamics of the yellow-necked mouse and, consequently, the multiannual dynamics of LCMV in this host species. Because antibodies against LCMV in Europe have been found in *A*. *flavicollis* mice from Italy (this study), Turkey (*13*), and Finland (*12*), masting-induced rodent dynamics may also affect the human incidence of LCMV in temperate areas of Europe. We are currently testing this hypothesis.

Our analysis also indicates clear seasonal variation; the number of infected mice increased as mouse density increased during the breeding season (Figure 4), although month did not appear to affect seroprevalence. This finding suggests that transmission between mice is not primarily between animals of different sex. Our results were consistent with those of Laakkonen et al. (*12*), who showed that sex of the mouse does not affect LCMV prevalence. However, our results suggest that weight and sex interact and show a correlation with antibody prevalence in host populations, as does age, so that heavier, older males are most likely to be LCMV positive. This result suggests horizontal transmission of LCMV by a mechanism that involves mainly males, such as infection by bite wounds inflicted during fighting (*32*–*35*). This hypothesis is supported by previous reports that male mice have a greater home range than females, and their home ranges overlap more than those of more territorial females (*29*,*36*,*37*). The fact that juveniles and subadults are less frequently infected than adults suggests that maturation and behavioral changes also play a role in virus transmission. Furthermore, although our results indicate that intraspecies transmission and maintenance of LCMV in *A*. *flavicollis* mice are dependent on social and spacing behavior in this species, other factors, such as genetic and physiologic variation at the individual, population or species level, could affect transmission.

Finally, for technical reasons, LCMV obtained from wild rodent species in Europe has not yet been isolated or sequenced. However, genetic characterization of arenaviruses is obviously crucial to the understanding of the ecology and epidemiology of LCMV and is one of our immediate goals.
